# Metabolically Healthy Obesity (MHO) vs. Metabolically Unhealthy Obesity (MUO) Phenotypes in PCOS: Association with Endocrine-Metabolic Profile, Adherence to the Mediterranean Diet, and Body Composition

**DOI:** 10.3390/nu13113925

**Published:** 2021-11-02

**Authors:** Luigi Barrea, Giovanna Muscogiuri, Gabriella Pugliese, Giulia de Alteriis, Annamaria Colao, Silvia Savastano

**Affiliations:** 1 Dipartimento di Scienze Umanistiche, Università Telematica Pegaso, Via Porzio, Centro Direzionale, Isola F2, 80143 Napoli, Italy; 2 Centro Italiano per la Cura e il Benessere del paziente con Obesità (C.I.B.O), Endocrinology Unit, Department of Clinical Medicine and Surgery, University Federico II, Naples, Via Sergio Pansini 5, 80131 Naples, Italy; giovanna.muscogiuri@gmail.com (G.M.); robiniapugliese@gmail.com (G.P.); colao@unina.it (A.C.); sisavast@unina.it (S.S.); 3 Unit of Endocrinology, Dipartimento di Medicina Clinica e Chirurgia, Federico II University Medical School of Naples, Via Sergio Pansini 5, 80131 Naples, Italy; dealteriisgiulia@gmail.com; 4 Cattedra Unesco “Educazione Alla Salute e Allo Sviluppo Sostenibile”, University Federico II, 80131 Naples, Italy

**Keywords:** metabolically healthy obesity (MHO), metabolically unhealthy obesity (MUO), PCOS, mediterranean diet, body composition, phase angle, nutritionist

## Abstract

Obesity and obesity-related low-grade inflammation are common findings in polycystic ovary syndrome (PCOS), the most common endocrine-metabolic disorder-affecting women in reproductive age. The terms metabolically healthy obese (MHO), and metabolically unhealthy obese (MUO) have been introduced to define individuals with obesity in whom cardio-metabolic risk factors are absent or present, respectively. To date, evidence investigating differences in body composition and adherence to the Mediterranean diet (MD) between MHO and MUO-PCOS women are lacking. Aim of this study was to better characterize the determinants of the metabolic health status in PCOS patients with obesity according to MHO and MUO phenotypes by evaluating endocrine-metabolic profile, inflammatory status, adherence to the MD, and body composition. The study population consisted of 94 treatment-naïve women with PCOS and obesity (BMI = 38.23 ± 6.62 kg/m^2^ and age = 24.12 ± 3.68 years). Compared PCOS MHO with PCOS MUO patients, the latter had higher levels of high-sensitivity C-reactive protein (hs-CRP) (*p* < 0.001), testosterone (*p* < 0.001), and insulin (*p* < 0.001), worse metabolic parameters, and higher Homeostatic Model Assessment of Insulin Resistance (HoMA-IR), Visceral Adiposity Index (VAI), and Fatty liver Index (FLI) (*p* < 0.001). Furthermore, PCOS MUO patients had lower adherence to the MD (*p* < 0.001) in spite of the same total energy intake (*p* = 0.102) as compared to PCOS MHO. The presence of MUO was associated with highest hs-CRP levels (OR = 1.49, *p* < 0.001), more severe hyperandrogenism and cardio-metabolic indices (*p* < 0.001). On the contrary, being PCOS MUO was associated with lower adherence to the MD (OR = 0.28, *p* < 0.001), and smaller PhAs (OR = 0.04, *p* < 0.001). Using a regression linear analysis model PREDIMED score entered at the first step (*p* < 0.001), followed by VAI (*p* < 0.001), and FLI (*p* = 0.032) in this analysis. At ROC analysis, a PREDIMED score of ≤4 (*p* < 0.001, AUC 0.926) could serve as a threshold for a significantly increased risk of presence the MUO-PCOS phenotype. To the best of our knowledge, this is the first study that characterized MHO and MUO-PCOS women on the basis of their adherence to the MD, body composition, and cardio-metabolic indices, providing evidence of the usefulness of adjunctive diagnostic parameters to better differentiate the MHO/MHO phenotypes in this cohort of PCOS patients with obesity.

## 1. Introduction

Obesity and obesity-related low-grade inflammation are common findings in polycystic ovary syndrome (PCOS), the most common endocrine-metabolic disorder-affecting women in reproductive age [1,2]. Considering the large body of evidence that clearly indicates the association of PCOS with increased long-term cardio-metabolic risks, the assessment of cardiovascular risk profile in women with PCOS is commonly recommended [3]. However, there is conflicting evidence on the relative role of obesity and PCOS status in this association [4]. Very recently, a systematic review of the literature reported that women with PCOS were more likely to be diagnosed with cardio-metabolic risk factors, although the extent to which increased cardio-metabolic risk was independent of obesity still remains to be resolved [5,6].

It is widely recognized that not all individuals with obesity will ultimately develop cardio-metabolic complications, as body mass index (BMI) *per se* does not take into account the increased risk associated with the heterogeneity of body fat distribution [6,7]. In particular, the pro-inflammatory state linked to the resultant ectopic lipid deposition and the adipose tissue dysfunction, which favors the insulin resistance in adipose tissue, skeletal muscle and liver, and features of the metabolic syndrome (MetS), represent emerging factors of global cardio-metabolic-risk [8]. Consequently, the terms metabolically healthy obese (MHO), and metabolically unhealthy obese (MUO) have been introduced to define individuals with obesity in whom cardio-metabolic risk factors are absent or present, respectively [9]. Currently, this categorization in obesity phenotypes still lacks standardized definitions [10]. As virtually all individuals with obesity had increased waist circumference (WC), the most used criteria to define MHO are based on the presence of ≥2 of the four diagnostic criteria for MetS according to the National Cholesterol Education Program Adult Treatment Panel III (NCEP ATP III) definition, with the exclusion of WC [11]. To further increase the debate surrounding the distinction between MHO and MUO, adjunctive criteria, such as insulin resistance and high-sensitivity C-reactive protein (hs-CRP), have also been proposed to better characterize the obesity phenotypes [10], and two cardio-metabolic indices, such as visceral adiposity index (VAI) and fatty liver index (FLI), both linked to the inflammatory pathways and considered early predictors of MetS, have demonstrated to represent predictive markers of the prognosis of MHO subjects and their MHO-to-MUO conversion [12,13]. In addition, lifestyle and body composition are generally not included in the current criteria. Data from an obese adult population of participants of the National Health and Nutrition Examination Survey III evidenced that the adherence to a Mediterranean dietary pattern was effective in reducing the mortality in the MHO phenotype, but not among the MUO phenotype, during a median follow-up of 18.5 years [14]. While, in a large sample of adults with obesity of both sex, MHO and MUO were characterized by differences in body composition measured with dual-energy X-ray absorptiometry (DXA) consistent between genders [15]. In clinical practice, body composition is commonly measured by bioelectrical impedance analysis (BIA), which is a non-invasive method for evaluation of body composition with a high agreement with DXA [16,17]. There are few studies investigating the clinical relevance of MHO and MHO phenotypes among PCOS women with obesity. Kim JY [18] reported that MHO-PCOS adolescent girls have worse anthropometric measurements, hormonal parameters, and metabolic features, and higher risk biomarkers for type 2 diabetes compared with MHO-PCOS unmatched or pair-matched for age and BMI [18]. In addition, Mu et al. [19] stratified a large-scale Chinese community population of women of reproductive age according to metabolic health and obesity status, and found that the prevalence of PCOS was increased among MHO women, suggesting that MHO status is most probably not a healthy condition [19]. Contrariwise, in women of reproductive age of Chinese ethnicity, Liang P et al. [20] did not found significant differences in the prevalence of PCOS with and without MS between MUO and BMI-matched MHO groups [20]. Therefore, the association of PCOS with MHO and MUO phenotypes still remains an open question. In addition, data investigating differences in body composition and adherence to the Mediterranean diet (MD) between MHO and MUO-PCOS women are lacking.

Aim of this study was to better characterize the determinants of the metabolic health status in PCOS women with obesity according to MHO and MUO phenotypes by evaluating endocrine-metabolic profile, adherence to the MD, inflammatory status, cardio-metabolic indices, and body composition.

## 2. Materials and Methods

This monocentric study was carried out in PCOS patients attending the Unit of Endocrinology, Obesity Unit at the Department of Clinical Medicine and Surgery, University “Federico II” of Naples from January 2018 to January 2020. This protocol has been approved by the Federico II Ethical Committee (n. 05/14) and carried out in accordance with the Declaration of Helsinki (Code of Ethics of the World Medical Association) for experiments that involved humans. All study participants signed informed consent after being clearly informed about the purpose of this research protocol.

This study included 94 patients with PCOS confirmed by an endocrinologist, coming from the same geographical area around Naples metropolitan area, Campania, Italy. To increase the homogeneity of the patient sample, we only included treatment-naïve patients. In particular, eligible patients were those with a diagnosis of PCOS classified by the ESHRE/ASRM (European Society for Human Reproduction and Embryology/American Society for Reproductive Medicine) diagnosis [21]. The diagnosis included the presence of two of the three features of hyperandrogenism (either clinical or biochemical (hirsutism by elevated Ferriman-Gallwey score or elevated testosterone or free androgen index, respectively), the presence of polycystic ovaries on ultrasound scan (ovarian volume >10 mL in at least one ovary or ≥12 follicles measuring 2–9 mm in diameter), and oligomenorrhea or amenorrhea (interval between two menstrual periods more than 35 days or no vaginal bleeding for at least six months, respectively). The degree of hirsutism was evaluated using Ferriman-Gallwey hirsutism scoring scale, which measures nine androgen sensitive areas in the body [22]. A Ferriman–Gallwey score ranging from 0 score (no excessive terminal hair growth visible) to four scores (extensive hair growth visible) for each body part evaluated and evaluates 11 different body parts. A maximum Ferriman-Gallwey score of 36 is possible but a Ferriman-Gallwey score of >7 or more was considered to be diagnostic of hirsutism [23]. Ferriman-Gallwey score was clinically evaluated by a single experienced endocrinologist, which was blinded to the design of the study to prevent biases. Other inclusion criteria were: pre-menopausal women who were with BMI ≥30.0 kg/m^2^, and aged 18–30 years.

The exclusion criteria were the following:

⮚ Patients with BMI <30.0 kg/m^2^;
⮚ Age < 18 years and >30 years;
⮚ Presence of menopause (defined as amenorrhea for ≥1 but <3 years and plasma follicle stimulating hormone concentrations elevated to the postmenopausal range or amenorrhea for ≥3 years);
⮚ Lactation or pregnancy in the past 6 months;
⮚ Oligomenorrhea, biochemical hyperandrogenemia, and/or hyperandrogenism due to secondary aetiologies including endocrine disorders as per the Endocrine Society Clinical Practice Guidelines and previous publications [24];
⮚ Pre-existing systemic or psychiatric disease;
⮚ Use of drugs that influence lipid or carbohydrate metabolism (anti-psychotics, metformin, oral contraceptive pills, anti-epileptics, fish oil, and statins) or current/occasional use of drugs that could influence fluid balance, including laxative, diuretics, and non-steroidal anti-inflammatory drugs;
⮚ Specific dietary patterns, including hypocaloric diet in the last three months;
⮚ Specific supplementation with dietary supplements that affect body weight, inflammation, or oxidation (such as antioxidants, vitamins or minerals);
⮚ Lack of underlying metabolic diseases (hypertension, cardiovascular diseases or previous cardiovascular events, anaemia, type 2 diabetes, and any other metabolic disease requiring a special dietary pattern or specific nutritional recommendations);
⮚ History of allergy to food belonging to the MD (including fish, nuts, and others);
⮚ Implanted pacemakers or defibrillators for the possibility of interference with the BIA-device;
⮚ Patients with skin damage in the specific area of application of the BIA electrodes.

The MetS was diagnosed according to the NCEP ATP III definition [25]. The WC criterion was not used because of its collinearity with BMI. Participants who met fewer than two of the following four criteria were considered MHO [11,26].

Lifestyle habits were determined in all study participants through a standard questionnaire [27,28,29,30].

After an overnight fast at least 8 h, in all PCOS patients were assesses the anthropometric measurements, in particular weight, height, and WC as previously reported [31,32,33]. BMI was calculated by weight (kg) and height squared (m^2^), in accordance with the WHO’s criteria [34].

As we reported earlier [35,36,37], the adherence to the MD was assessed by the PREvention with MEDiterranean Diet (PREDIMED) questionnaire, a brief 14-item questionnaire to able to capture the adherence to the MD in adults at high cardiovascular risk and obesity [38]. As we have already fully reported in previous studies [39,40,41], the total energy intake was obtained by a face-to-face interview that a qualified nutritionist that who administered a seven-day food records. According of these records, the nutritionist calculated the total energy intake [42].

The samples were collected after at least 8 h an overnight fast, in the morning between 8 and 10 am, and stored at −80 °C until being processed. All biochemical analyses were performed with a Roche Modular Analytics System in the Central Biochemistry Laboratory of our Institution. A hs-CRP levels were categorised according to cardiovascular risk category to the American Heart Association/Centers for Disease Control (hs-CRP levels ≥3 mg/L as high risk of cardiovascular disease) [43].

Cardio-metabolic indices, including homeostatic model assessment-insulin resistance (HoMA-IR) [44], visceral adiposity index (VAI) [45,46], and fatty liver index (FLI) [47] have been calculated.

According to the European Society for Clinical Nutrition and Metabolism (ESPEN) [48], body composition measurements using a BIA phase-sensitive system (BIA 101, RJL Akern Bioresearch, Florence, Italy, 800 µA current at a single frequency of 50 kHz), as previously reported [49,50,51]. Phase angle (PhA) (°, degrees) was obtained by the formula: Xc/R*(180/π) [52]. See details in Supplementary Material.

### Statistical Analysis

The sample size was calculated carried out using the Sample Size Calculator program as reported in several previous studies [53,54,55]. In particular, as study group design two independent study groups were considered, PCOS MHO vs. PCOS MUO. The differences of means  ±  standard deviation (SD) of testosterone levels were used as the primary endpoint (29.21 ± 9.44 vs. 42.33 ± 9.64 ng/dL in PCOS MHO vs. PCOS MUO, respectively). Considering a type I (alpha) error of 0.05 (95%), a type II (beta) of 0.05, and a power size of 95%, the minimum number of cases required in MUO and MHO group was 13. The calculation of sample size and power were performed using Sample Size Calculator Clinical Calc [56]. Data were collected and analyzed using the SPSS v.23.0 statistical package. The data distribution was evaluated by Kolmogorov-Smirnov test and the abnormal data (BMI, WC, PREDIMED score, total energy intake, hs-CRP levels, testosterone, insulin, fasting glucose, total cholesterol, HDL-cholesterol, LDL-cholesterol, triglycerides, ALT, AST, γGT, HoMA-IR, VAI, FLI, MetS (number parameter), Ferriman-Gallwey score, R, Xc, and PhA) were normalized by logarithm. Results have been described as mean ± SD or percentage/number. Differences according to MHO/MUO phenotype were analyzed by Student’s unpaired *t*-test. The chi square (χ^2^) test was used to evaluate the differences in the frequency distribution of smoking, physical activity, BMI classes, three different categories of adherence to the MD, cut-offs of hs-CRP, HoMA-IR, VAI, and FLI, and presence/absence of MetS. Proportional Odds Ratio (OR) models, 95% Interval Confidence (IC), and R^2^, were used to assess the associations among MHO/MUO phenotype with anthropometric measurements, nutritional, inflammatory, hormonal, and metabolic parameters, cardio-metabolic indices, clinical hyperandrogenism, and body composition. A multiple linear regression analysis model (stepwise method) expressed as R^2^, beta (β), and *t*, with the presence of the MUO-PCOS phenotype as dependent variables were used to estimate the predictive value of WC, hs-CRP levels, PREDIMED score, cardio-metabolic-indices, Ferriman-Gallwey score, testosterone, and PhA. Receiver operator characteristic (ROC) curve analysis was performed to determine the sensitivity and specificity, the area under the curve (AUC) as a measure of the accuracy of the test, as well as cut-off value of the adherence to the MD. The optimal cutoff threshold was determined at the point on the ROC curve at which (sensitivity + specificity − 100%) was maximal. Data were analyzed using the SPSS Software (PASW Version 21.0, SPSS Inc., Chica-go, IL, USA) and MedCalc^®^ package (Version 12.3.0 1993- 2012 MedCalc Software bvba—MedCalc Software, Mariakerke, Belgium).

## 3. Results

The study population consisted of 94 patients with PCOS. All patients completed the study protocol, including the anthropometric measurements, nutritional assessment, and BIA measurements. Table 1 reports all parameters analysed in this study.

Table 2 shows the percentages of categorical variables analysed in this study. More than 50% of the participants were smokers and more than 65% were sedentary. The highest percentage of participants had grade II obesity (44.7%). None of the participants reported having high adherence to the MD. The 34.0% of patients had hs-CRP levels ≥3 mg/L representative of a high risk of cardiovascular disease. More than 60% of patients had all three cardio-metabolic indices evaluated in this study greater than the cutoffs values. MetS was present in 67% of patients; Table 2.

Table 3 reports the differences in lifestyle habits, anthropometric measurements, nutritional parameters, hs-CRP levels, hormonal, biochemical, and metabolic parameters, cardio-metabolic indices and metabolic syndrome, ferriman-Gallwey score, and body composition measurements according to metabolically healthy versus metabolically unhealthy. Comparing PCOS patients MHO with MUO patients, the latter had the highest BMI and WC values (*p* < 0.001), higher levels of inflammation (*p* < 0.001), testosterone (*p* < 0.001), and insulin (*p* < 0.001), worst metabolic parameters, highest cardio-metabolic indices (*p* < 0.001), multiple clusters of the MetS (*p* < 0.001), and higher Ferriman-Gallwey score values (*p* < 0.001). Furthermore, PCOS MUO patients had lower adherence to the MD (*p* < 0.001) for the same total energy intake (*p* = 0.102) compared to MHOs.

The differences in categorical study parameters according to metabolically healthy versus metabolically unhealthy were summarized in Table 4. The highest percentage of PCOS MUO patients had grade III obesity (*p* < 0.001). Furthermore, PCOS MUO patients compared to MHOs had the highest percentage of low adherence to the MD (*p* < 0.001), the highest percentage of hs-CRP levels > cut-off (*p* = 0.001), and the highest percentage of all three cardio-metabolic indices evaluated in this study, including HoMA-IR (*p* < 0.001), VAI (*p* = 0.001), and FLI (*p* = 0.030). MetS was diagnosed in all 40 PCOS MUO patients (*p* < 0.001).

To assess the association of PCOS patients MHO/MUO with all the continuous variables including in this study, we performed a bivariate proportional OR model with MHO/MUO as categorical variable. The results of the OR model are reported in Table 5. The presence of MUO was associated with highest BMI (OR = 1.41, *p* < 0.001), WC (OR = 1.11, *p* < 0.001), hs-CRP levels (OR = 1.49, *p* < 0.001), hormonal parameters (*p* < 0.001), metabolic parameters, cardio-metabolic indices (*p* < 0.001), and Ferriman-Gallwey score (OR = 1.21, *p* < 0.001). On the contrary, being MUO was associated with lower adherence to the MD (OR = 0.28, *p* < 0.001), and smaller PhAs (OR = 0.04, *p* < 0.001).

To assess the relative prognostic value of the WC, PREDIMED score, hs-CRP levels, hormonal parameters, cardio-metabolic indices, and PhA to predict the presence of the MUO-PCOS phenotype, we performed a regression linear analysis model including these parameters. Using this model, PREDIMED score entered at the first step (*p* < 0.001), followed by VAI (*p* < 0.001), and FLI (*p* = 0.032); Table 6.

**Figure 1:**
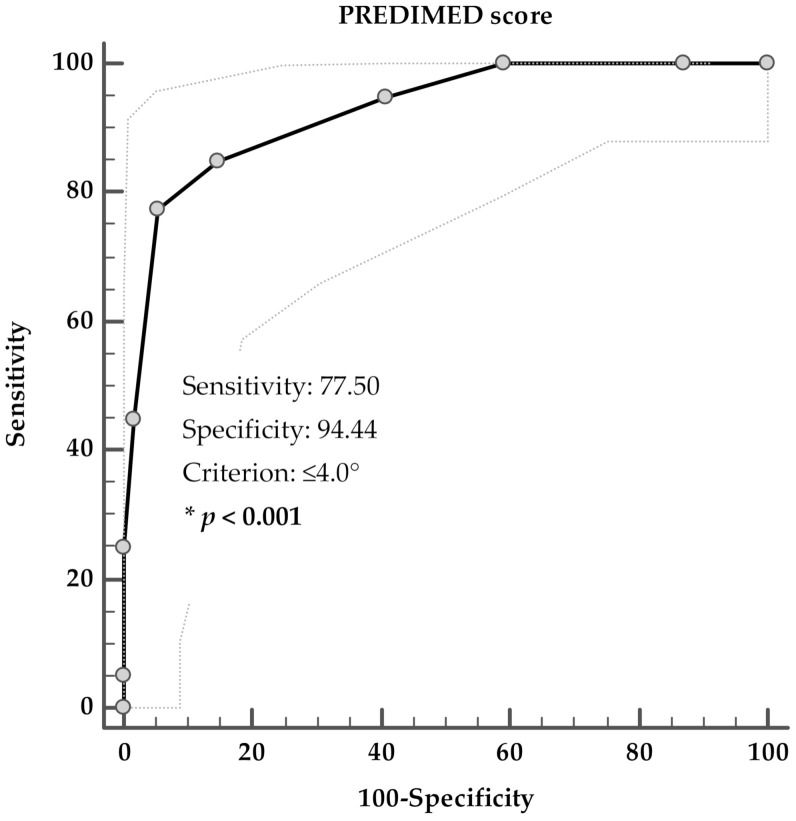
ROC for predictive values of PREDIMED score in detecting the presence of PCOS MUO phenotype. * A *p* value in bold type denotes a significant difference (*p* < 0.001).

A ROC analysis was performed to determine the cut off value of the adherence to the MD predictive of the presence of the PCOS MUO phenotype. A score of PREDIMED of ≤4 (*p* < 0.001, AUC 0.926, standard error 0.027, 95% CI 0.874 to 0.978) could serve as a threshold for a significantly increased risk of presence the MUO-PCOS phenotype; Figure 1.

## 4. Discussion

In this study, we investigated the differences in the nutritional status and cardio-metabolic indices in a cohort of treatment-naïve women with PCOS classified as MHO and MUO according to MetS criteria. As expected, MUO-PCOS patients presented both worse endocrine and metabolic profiles, with higher levels of testosterone, higher values of Ferriman-Gallwey score and of HoMA-IR, and higher levels of hs-CRP, as compared to their MHO-counterpart. As novel findings, we better characterized the metabolic risk profile as we evidenced that MUO-PCOS patients presented also lower adherence to the MD, higher values of VAI and FLI, and smaller PhAs as compared to their MHO-counterpart. In particular, the adherence to the MD, VAI, and FLI were the factors that exerted the most powerful influence on the presence of the MUO-PCOS phenotype. A PREDIMED score ≤4 was found as the most sensitive and specific cut-point value to predict the presence of the MUO-PCOS phenotype. To the best of our knowledge, this is the first study that characterized MHO and MUO-PCOS patients, including the nutritional status and two cardio-metabolic indices that are considered early predictors of MetS. On this basis, we suggested the usefulness of adjunctive diagnostic parameters to better differentiate the MHO/MHO phenotypes in this cohort of PCOS patients with obesity.

The distinction between MHO and MUO have been introduced to define individuals with obesity in whom cardio-metabolic risk factors are absent or present, respectively. Currently, the most used criteria to define MHO are based on the presence of ≤2 of the four diagnostic criteria for MetS according to the NCEP ATP III definition [11]. Nevertheless, a growing body of evidence indicated that MHO individuals are not ‘healthy’, as they are at higher risk of atherosclerotic cardiovascular disease, hearth failure, and respiratory diseases and some cancers compared with non-obese individuals with a normal metabolic profile [57,58]. Therefore, the inclusion of adjunctive criteria, such as insulin resistance and hs-CRP have been proposed to better characterize the different metabolic risk profile between MHO/MUO phenotypes, either in individuals with obesity [7,9,10] or in PCOS patients [6,18]. In addition, differences in dietary pattern, such as adherence to the MD [14], and body composition [15], have been reported to further influence the metabolic risk profile of the two obesity phenotypes. Nevertheless, the association of the metabolic risk profile of MHO/MUO with the nutritional status and cardio-metabolic indices in adult PCOS with obesity has not been investigated previously.

Our findings on the differences in metabolic risk profile of MHO/MUO phenotypes in adult women with PCOS are line with the study of Kim JY et al. (2016) in adolescent girls with PCOS [18]. These Authors investigated the body composition, metabolic, hormonal and cardiovascular characteristics of MHO vs. MUO in adolescent girls with PCOS and found that adolescent MUO-PCOS had worse body composition, hormonal and metabolic features as compared with MHO-PCOS unmatched or pair-matched for age and BMI [18]. Also, Mu L et al. (2018) [19] more recently investigated the metabolic risk profile in a very large survey of PCOS women of reproductive age and found that MUO-PCOS group had worse metabolic and endocrine profile as compared with the MHO-PCOS group [19]. In this study, however, the Authors found no significant difference in BMI between the two groups of PCOS with obesity, but their BMI values were lower than those in our population sample and [19]. In addition, in neither of these two studies the nutritional status and cardio-metabolic indices were included to characterize the metabolic risk profile of PCOS MHO and MUO.

VAI and FLI are two cardio-metabolic indices linked to the inflammatory pathways and early predictors of MetS [59,60,61]. In particular, VAI is an index based on a combination of anthropometric and metabolic parameters that is highly correlated with visceral adiposity measured by magnetic resonance imaging and expression of dysfunctional adipocytes [62]. FLI is a well-established non-invasive composite proxy to assess liver steatosis [47]. Both VAI and FLI have well demonstrated to represent predictive markers of the prognosis not only among MHO subjects [12,13], but also in PCOS women with obesity [59,60,61]. More specifically, de Medeiros SF [63] found that VAI was the strongest predictor of MetS in both obese and non-obese PCOS women, suggesting its usefulness in clinical practice to detect the risk for MetS and to early address adequate therapeutical interventions in women with PCOS, particularly in women with obesity. However, in this study the Authors did not distinguish PCOS women with obesity according to MHO/MUO phenotypes. In our study, VAI values were higher in MUO-PCOS than in MHO-counterpart. In that, our findings suggested a possible use of VAI as distinctive marker of MUO presence among women with PCOS. As novel finding, we found that also FLI was higher in MUO-PCOS than in MHO-counterpart [63]. Liang P et al. (2017) [20] previously investigated the prevalence of liver steatosis evaluated by FLI in PCOS women with obesity of reproductive age and reported that level of FLI were lower in MHO as compared with MUO-PCOS, and were associated with higher liver/spleen ratio as marker of less fat accumulation in the liver. Our findings on the differences in VAI and FLI between MHO/MUO PCOS phenotypes in line with these two studies. However, we further extended the knowledge on the differences in VAI and FLI between MHO/MUO PCOS phenotypes, as we found that both VAI and FLI well predicted the MUO presence, although these variables entered after the adherence to the MD. Of interest, we have previously reported that higher levels of liver/spleen index were significantly associated with the low-grade chronic inflammation in PCOS women with high plasma levels of bisphenol A, one of the most common endocrine disruptors involved in the obesity pathogenesis and the alterations of several metabolic functions, including inflammatory pathways [64].

There is a general consensus that lifestyle modifications for PCOS, including physical activity and diet, are currently the mainstay to improve the metabolic deregulation related to this condition [65,66,67]. In particular, it is well established that the MD exerts beneficial effects on inflammation hyperandrogenism and cardiometabolic risk profile in PCOS. MD, characterized by high consumption of extra virgin olive oil, fish, vegetables, legumes, whole-grain products, fruits, and nuts, has been demonstrated to exert relevant anti-inflammatory and anti-oxidative effects on different clinical settings, including inflammatory and immune processes [68], psoriasis [69], acne [70], hidradenitis suppurativa [71], breast cancer [72,73], endocrine dysfunction [74,75], sleep disturbance [76,77], or menopause [78,79]. In particular, MD provides a significant source of antioxidant vitamins thus decrease inflammation and oxidative stress [80]. Park YM (2016) reported the differential beneficial association of MD on mortality risk reduction in MHO and not in MUO phenotype in individuals with obesity [14]. We previously reported that PCOS women had lower adherence to the MD as compared with BMI-matched controls, with an inverse association among the adherence to MD, BMI, and the clinical severity of the disease [81]. Accordingly, Cutillas-Tolín A et al. (2021) [82] found that the women with PCOS and a higher adherence scores to the MD had a lower BMI. In our study, we confirmed that a low adherence to the MD was very common among MUO-PCOS, as up to 85% of MUO-PCOS had a PREDIMED score <5. As novel finding, we evidenced that the adherence to the MD was also different in MHO/MUO phenotypes, being a PREDIMED score ≤4 the best predictor of the MUO presence. In addition, in line with the well-known anti-inflammatory and anti-oxidant properties of the MD [80] we found that the MUO-PCOS with a low adherence to the MD presented also high hs-CPR levels and small PhAs, two distinctive markers of inflammation in this study. CRP, a commonly used biomarker of meta-inflammation [83], has recently been included among criteria to define the obesity phenotypes [10]. PhA, a non-invasive bioimpedance marker, reflects cell membrane integrity and size, and it is used as a marker of body cell mass [84]. Altered measurements of the PhA are associated with inflammatory markers [85], are commonly used in the nutritional screening of several diseases, are an important independent predictor of mortality [86], and might represent an easy detectable marker of inflammation that avoid the collection of blood sampling and expensive biochemical assays [87]. Correlations have been found between increased levels of CRP and other markers of inflammation in the PCOS women compared with age- and BMI-matched controls [1]. Nevertheless, when CRP levels were analysed considering the MHO/MUO PCOS phenotype in the previously cited study by Liang P, no differences were found between the two groups [20].

As possible translational applications, in line with the current literature, our study suggested that the adherence to the MD, the determination of the PhA, as easy detectable marker of inflammation, and VAI and FLI, as early predictors of the MetS, should be considered as adjunctive criteria in the characterization of the metabolic risk profile in MHO/MUO-PCOS phenotypes. In particular, by using the PREDIMED score, which is a short, valid and easy questionnaire, a cut-off value score ≤4 could serve as predictor of the risk of MHO-to-MUO conversion among PCOS patients with obesity in the clinical practice. The association of the low adherence to the MD with worse endocrine and metabolic profiles in PCOS patients with obesity let us to speculate that the anti-inflammatory and anti-oxidant properties of the MD, through the improvements in the hyperandrogenism, cardio-metabolic indices, and pro-inflammatory status, could act as synergistic mechanism to prevent MHO-to-MUO conversion among PCOS patients with obesity.

Limitations of this study warrant some considerations. First, the cross-sectional design of this study does not allow for causal inference and prospective on the role of the adherence to the MD in the prediction of the MUO phenotype in PCOS patients. Therefore, the proposed cut-off point of PREDIMED score for identifying the MUO phenotype should be validated by further clinical trials. Second, the sample size is relatively small. However, the sample size was calculated by using 95% statistical power that assured an adequate power to detect statistical significance on the results. Third, we did not analyse other inflammatory markers. Nevertheless, it is widely reported that CRP represents the most studied inflammatory biomarker in different pathologic processes [88]. In addition, we used hs-CRP method, which allows an acceptable precision level of <0.2 mg/L.

Nevertheless, this study also has several strengths. First, the accurate characterization of the PCOS study population by a trained team of Endocrinologists and Nutritionists. In particular, to increase the homogeneity of the PCOS sample, all PCOS patients included were only naïve to treatment and came from the same geographical area, thus possibly sharing overall similar eating habits and food availability. Second, to further minimize the inter-operator variability, a single nutritionist performed and interpreted BIA-parameters and evaluated the adherence to the MD and the total energy intake of both MHO and MUO PCOS patients. In particular, either adherence to the MD evaluated by PREDIMED questionnaire, which was recently validated in different Mediterranean countries, including Italy [89], or the total energy intake evaluated by the seven-day food record, the gold standard among food frequency questionnaire, were not self-reported, but face-to-face administered, to reduce any bias related to the filling in of these questionnaires.

## 5. Conclusions

The adherence to the MD, which can be evaluated by a short, valid and easy questionnaire, represented the major determinant of the MUO phenotype in PCOS patients. The results of this study underline the importance of assessment of the nutritional status to better address PCOS patients with obesity to a personalized, intensive weight-loss program. Growing cooperation between Endocrinologists and Nutritionists confirmed to be the better synergistic combination in the complex clinical management of PCOS patients with obesity.

## Figures and Tables

**Table 1:** Parameters evaluated in the study in PCOS patients.

Parameters	PCOS Patients n = 94 Mean ± SD
Age (years)	24.12 ± 3.68
Anthropometric measurements	
BMI (kg/m^2^)	38.23 ± 6.62
WC (cm)	117.66 ± 19.53
Nutritional parameters	
PREDIMED score	5.56 ± 2.25
Total energy intake (kcal)	2636.09 ± 415.77
Inflammatory parameter	
hs-CRP levels (mg/L)	3.61 ± 4.19
Hormonal parameters	
Testosterone (ng/dL)	54.79 ± 11.51
Insulin (μU/mL)	19.17 ± 14.99
Metabolic parameters	
Fasting glucose (mg/dL)	104.94 ± 15.21
Total cholesterol (mg/dL)	200.55 ± 38.91
HDL-cholesterol (mg/dL)	37.61 ± 11.06
LDL-cholesterol (mg/dL)	129.27 ± 41.15
Triglycerides (mg/dL)	168.38 ± 50.79
ALT (U/L)	41.74 ± 19.10
AST (U/L)	41.31 ± 18.37
γGT (U/L)	39.93 ± 21.31
Cardio-metabolic indices	
HoMA-IR	5.32 ± 4.59
VAI	4.51 ± 2.44
FLI	87.60 ± 14.04
Metabolic Syndrome	
MetS (number parameter)	3.13 ± 1.22
Clinical Hyperandrogenism	
Ferriman-Gallwey score	19.61 ± 8.18
Body composition	
R (Ω)	460.39 ± 84.64
Xc (Ω)	43.79 ± 9.47
PhA (°)	5.47 ± 0.75

PCOS, Polycystic Ovarian Syndrome; BMI, Body Mass Index; WC, Waist Circumference; PREDIMED, PREvención con DIetaMEDiterránea; hs-CRP, high-sensitivity C-reactive Protein; HDL, High-density Lipoprotein; LDL, Low-Density Lipoprotein; ALT, Alanine Transaminase; AST, Aspartate Aminotransferase; γGT; γ-Glutamyltransferase; HoMA-IR, Homeostasis model assessment insulin resistance; VAI, Visceral Adiposity Index; FLI; Fatty Liver Index; MetS, Metabolic Syndrome; R, Resistance; Xc, Reactance; PhA, Phase angle; SD, standard deviation.

**Table 2:** Categorical variables analysed in this study.

Parameters	PCOS Patients n = 94 (*n*, %)
Lifestyle Habits	
Smoking (yes)	52, 55.3%
Physical activity (yes)	31, 33.0%
BMI	
Grade I obesity	34, 36.2%
Grade II obesity	42, 44.7%
Grade III obesity	18, 19.1%
Adherence to the MD	
Low-adherence to the MD	42, 44.7%
Average-adherence to the MD	52, 55.3%
High-adherence to the MD	0, 0%
Inflammatory parameter	
hs-CRP levels > cut-off	32, 34.0%
Cardio-metabolic indices	
HoMA-IR > cut-off	57, 60.6%
VAI > cut-off	71, 75.5%
FLI > cut-off	86, 91.5%
Metabolic Syndrome	
MetS (presence)	63, 67.0%

PCOS, Polycystic Ovarian Syndrome; BMI, Body Mass Index; MD, Mediterranean diet; hs-CRP, high-sensitivity C-reactive Protein; HoMA-IR, Homeostasis model assessment insulin resistance; VAI, Visceral Adiposity Index; FLI; Fatty Liver Index; MetS, Metabolic Syndrome.

**Table 3:** Differences in study parameters according to metabolically healthy versus metabolically unhealthy.

Parameters	PCOS MHO n = 54 Mean ± SD	PCOS MUO n = 40 Mean ± SD	* p-Value
Age (years)	23.83 ± 3.67	24.50 ± 3.68	0.388
Anthropometric measurements			
BMI (kg/m^2^)	34.85 ± 3.49	42.80 ± 7.11	**<0.001**
WC (cm)	106.74 ± 12.34	132.42 ± 17.72	**<0.001**
Nutritional parameters			
PREDIMED score	6.91 ± 1.44	3.68 ± 1.53	**<0.001**
Total energy intake (kcal)	2575.63 ± 388.52	2717.73 ± 441.79	0.102
Inflammatory parameter			
hs-CRP levels (mg/L)	1.86 ± 1.43	5.97 ± 5.39	**<0.001**
Hormonal parameters			
Testosterone (ng/dL)	49.21 ± 9.44	62.33 ± 9.64	**<0.001**
Insulin (μU/mL)	10.86 ± 10.42	30.38 ± 12.80	**<0.001**
Metabolic parameters			
Fasting glucose (mg/dL)	96.24 ± 9.36	116.70 ± 13.63	**<0.001**
Total cholesterol (mg/dL)	188.76 ± 33.10	216.48 ± 40.86	**0.001**
HDL-cholesterol (mg/dL)	42.46 ± 11.71	31.05 ± 5.34	**<0.001**
LDL-cholesterol (mg/dL)	117.24 ± 35.33	145.51 ± 43.26	**0.001**
Triglycerides (mg/dL)	145.27 ± 40.43	199.58 ± 46.84	**<0.001**
ALT (U/L)	35.45 ± 16.74	49.21 ± 17.67	**0.002**
AST (U/L)	36.26 ± 15.27	49.15 ± 21.35	**<0.001**
γGT (U/L)	26.94 ± 12.64	57.48 ± 17.79	**<0.001**
Cardio-metabolic indices			
HoMA-IR	2.65 ± 2.73	8.92 ± 4.12	**<0.001**
VAI	3.22 ± 1.60	6.25 ± 2.31	**<0.001**
FLI	80.28 ± 14.18	97.49 ± 4.74	**<0.001**
Metabolic Syndrome			
MetS (number parameter)	2.24 ± 0.75	4.24 ± 0.47	**<0.001**
Clinical Hyperandrogenism			
Ferriman-Gallwey score	15.76 ± 7.17	24.80 ± 6.45	**<0.001**
Body composition			
R (Ω)	458.15 ± 82.52	463.43 ± 88.37	0.767
Xc (Ω)	47.11 ± 9.51	39.30 ± 7.41	**<0.001**
PhA (°)	5.91 ± 0.53	4.89 ± 0.59	**<0.001**

PCOS, Polycystic Ovarian Syndrome; MHO, metabolically healthy obesity; MUO; metabolically unhealthy obesity; SD, standard deviation; BMI, Body Mass Index; WC, Waist Circumference; PREDIMED, PREvención con DIetaMEDiterránea; hs-CRP, high-sensitivity C-reactive Protein; HDL, High-density Lipoprotein; LDL, Low-Density Lipoprotein; ALT, Alanine Transaminase; AST, Aspartate Aminotransferase; γGT; γ-Glutamyltransferase; HoMA-IR, Homeostasis model assessment insulin resistance; VAI, Visceral Adiposity Index; FLI; Fatty Liver Index; MetS, Metabolic Syndrome; R, Resistance; Xc, Reactance; PhA, Phase angle. * A *p* value in bold type denotes a significant difference (*p* < 0.05).

**Table 4:** Differences in categorical study parameters according to metabolically healthy versus metabolically unhealthy.

Parameters	PCOS MHO n = 54 (*n*, %)	PCOS MUO n = 40 (*n*, %)	χ^2^	* p-Value
Lifestyle Habits				
Smoking (yes)	27, 50.0%	25, 62.5%	0.99	0.320
Physical activity (yes)	21, 38.9%	10, 25.0%	1.43	0.232
BMI				
Grade I obesity	30, 55.6%	4, 10.0%	18.73	**<0.001**
Grade II obesity	21, 38.9%	21, 52.5%	1.22	0.270
Grade III obesity	3, 5.6%	15, 37.5%	13.15	**0.001**
Adherence to the MD				
Low-adherence to the MD	8, 14.8%	34, 85.0%	43.00	**<0.001**
Average-adherence to the MD	46, 85.2%	6, 15.0%		
High-adherence to the MD	0, 0%	0, 0%		
Inflammatory parameter				
hs-CRP levels > cut-off	10, 18.5%	22, 55.0%	12.04	**0.001**
Cardio-metabolic indices				
HoMA-IR > cut-off	17, 31.5%	40, 100.0%	42.37	**<0.001**
VAI > cut-off	32, 59.3%	39, 97.5%	16.17	**0.001**
FLI > cut-off	46, 85.2%	40, 100.0%	4.71	**0.030**
Metabolic Syndrome				
MetS (presence)	23, 42.6%	40, 100.0%	31.71	**<0.001**

PCOS, Polycystic Ovarian Syndrome; MHO, metabolically healthy obesity; MUO; metabolically unhealthy obesity; BMI, Body Mass Index; MD, Mediterranean diet; hs-CRP, high-sensitivity C-reactive Protein; HoMA-IR, Homeostasis model assessment insulin resistance; VAI, Visceral Adiposity Index; FLI; Fatty Liver Index; MetS, Metabolic Syndrome. * A *p* value in bold type denotes a significant difference (*p* < 0.05).

**Table 5:** Bivariate OR model to assess the association of PCOS patients MHO/MUO with all continuous variables including in this study.

PCOS Patients MHO/MUO n = 94				
Parameters	OR	* p-Value	95% IC	R^2^
Anthropometric measurements				
BMI (kg/m^2^)	1.41	**<0.001**	1.20–1.66	0.37
WC (cm)	1.11	**<0.001**	1.07–1.15	0.40
Nutritional parameters				
PREDIMED score	0.28	**<0.001**	0.17–0.45	0.51
Total energy intake (kcal)	1.00	0.105	1.00–1.00	0.03
Inflammatory parameter				
hs-CRP levels (mg/L)	1.49	**<0.001**	1.19–1.86	0.25
Hormonal parameters				
Testosterone (ng/dL)	1.14	**<0.001**	1.08–1.20	0.30
Insulin (μU/mL)	1.13	**<0.001**	1.07–1.18	0.38
Metabolic parameters				
Fasting glucose (mg/dL)	1.16	**<0.001**	1.09–1.23	0.42
Total cholesterol (mg/dL)	1.02	**0.001**	1.00–1.03	0.12
HDL-cholesterol (mg/dL)	0.86	**<0.001**	0.79–0.92	0.29
LDL-cholesterol (mg/dL)	1.02	**0.002**	1.00–1.03	1.12
Triglycerides (mg/dL)	1.03	**<0.001**	1.01–1.04	0.27
ALT (U/L)	1.04	**0.002**	1.01–1.07	0.11
AST (U/L)	1.05	**0.001**	1.02–1.06	0.13
γGT (U/L)	1.15	**<0.001**	1.09–1.21	0.49
Cardio-metabolic indices				
HoMA-IR	1.55	**<0.001**	1.31–1.83	0.42
VAI	2.24	**<0.001**	1.63–3.08	0.37
FLI	1.28	**<0.001**	1.15–1.43	0.45
Clinical Hyperandrogenism				
Ferriman-Gallwey score	1.21	**<0.001**	1.12–1.31	0.30
Body composition				
PhA (°)	0.04	**<0.001**	0.01–0.15	0.44

PCOS, Polycystic Ovarian Syndrome; MHO, metabolically healthy obesity; MUO; metabolically unhealthy obesity; BMI, Body Mass Index; WC, Waist Circumference; PREDIMED, PREvención con DIetaMEDiterránea; hs-CRP, high-sensitivity C-reactive Protein; HDL, High-density Lipoprotein; LDL, Low-Density Lipoprotein; ALT, Alanine Transaminase; AST, Aspartate Aminotransferase; γGT; γ-Glutamyltransferase; HoMA-IR, Homeostasis model assessment insulin resistance; VAI, Visceral Adiposity Index; FLI; Fatty Liver Index; MetS, Metabolic Syndrome; R, Resistance; Xc, Reactance; PhA, Phase angle; OR, Odds Ratio; IC, Interval Confidence. * A *p* value in bold type denotes a significant difference (*p* < 0.05).

**Table 6:** Regression linear analysis to estimate the predictive value of WC, PREDIMED score, hs-CRP levels, hormonal parameters, cardio-metabolic indices, and PhA on the presence of the MUO-PCOS phenotype.

Parameters	Multiple Regression Analysis			
	R^2^	*β*	t	* p Value
**PREDIMED score**	0.539	−0.738	−0.48	**<0.001**
**VAI**	0.591	0.288	3.55	**0.001**
**FLI**	0.619	0.189	2.18	**0.032**
Variable excluded: WC, hs-CRP levels, Ferriman-Gallwey score, testosterone, HoMA-IR, PhA				

PREDIMED, PREvención con DIetaMEDiterránea; VAI, Visceral Adiposity Index; FLI; Fatty Liver Index; WC, Waist Circumference; hs-CRP, high-sensitivity C-reactive Protein; HoMA-IR, Homeostasis model assessment insulin resistance; PhA, Phase angle. * A *p* value in bold type denotes a significant difference (*p* < 0.05).

## Data Availability

Results attained in this study are included in the manuscript. Individual data are not publicly available due to ethical restrictions.

## Supplementary Materials

The following are available online at https://www.mdpi.com/article/10.3390/nu13113925/s1, Supplementary Material.
