# Supplementary material for: Metabolically Healthy Obesity (MHO) vs. Metabolically Unhealthy Obesity (MUO) Phenotypes in PCOS: Association with Endocrine-Metabolic Profile, Adherence to the Mediterranean Diet, and Body Composition
Source: Nutrients. 2021 Nov 2;13(11):3925. doi: 10.3390/nu13113925 (PMC8624317; doi:10.3390/nu13113925)
Supplement: Supplementary file 1 [file nutrients-13-03925-s001.zip › nutrients-1422301-supplementary.pdf]

Metabolic Syndrome (MetS) and MUO/MHO phenotypes

The MetS was present if three or more of the following five criteria are met: WC 88 cm, blood pressure ≥ 130/85 mmHg, fasting triglycerides level ≥ 150 mg/dL, fasting high-density lipoprotein (HDL) cholesterol level ≤50 mg/dL, and fasting glucose ≥ 100 mg/dL. Participants who met fewer than two of the following four criteria were considered MHO: (i) a systolic blood pressure ≥130 mmHg and/or a diastolic blood pressure ≥85 mmHg, or on antihypertensive treatment; (ii) fasting triglycerides level ≥150 mg/dL; (iii) fasting plasma glucose ≥100 mg/dL; and (iiii) fasting HDL cholesterol level ≤50 mg/dL.

Lifestyle habits

Considering smoking habits, PCOS patients were classified as current smokers (smoking at least one cigarette *per* day) and in non-current smokers (non-smokers or who had stopped for 2 years), as we reported earlier. Similarly, physical activity levels were classified as: inactive or active patients, based on the minutes of daily physical activity practiced *per* day. In particular, PCOS patients who practiced at least 30 min of daily aerobic physical activity were classified as active as already reported in other evidence

Anthropometric measurements and blood pressure

The weight was measurement with a calibrated balance beam scale with the nearest 50 g (Seca 711; Seca, Hamburg, Germany). The height was assessed used a wall-mounted stadiometer to the nearest 1 cm. Both for the measurement of weight and height, all participants wore light clothing and were without shoes when measuring. BMI was calculated by weight (kg) and height squared (m^2) and PCOS participants were classified into three BMI classes: grade I obesity (BMI= 30.0–34.9 kg/m^2), grade II obesity (BMI= 35.0–39.9 kg/m^2), and grade III obesity (BMI= ≥40.0 kg/m^2, respectively). According to the NCHS, WC was obtained using a non-stretchable measuring tape to the closest 0.1 cm at the narrowest point or to the nearest 0.1 cm at umbilical level in patients where no narrowest point of the waist was visible or PCOS patients with grade III obesity.

Nutritional parameters: adherence to the MD and total energy intake

In detail, scores of one (Yes) and zero (No), were assigned for each item of the PREDIMED questionnaire. From the sum of the 14-items of the PREDIMED questionnaire, the PREDIMED score was calculated. In accordance with the PREDIMED score, PCOS patients have been divided into the categories of adherence to the MD: (PREDIMED score ≥ 10 = high adherence to the MD, 6≤PREDIMED score ≤9 = average adherence to the MD, and PREDIMED score ≤ 5 = low adherence to the MD).

Assay Methods: hormonal and metabolic profile

Serum testosterone levels was measured by chemiluminescent enzyme immunoassay (Immulite 2000, Diagnostic Products Corp). The fasting insulin levels were measured by a solid-phase chemiluminescent enzyme immunoassay using commercially available kits (Immulite Diagnostic Products Co., Los Angeles, CA, USA). The intra-assay coefficients of variations (CV) of

serum testosterone (n. v. 7-59 ng/dL) and fasting insulin levels (n. v. 1-20 μU/mL) were <7% for both assays. HDL cholesterol and Low-Density Lipoprotein (LDL) cholesterol were determined by a direct method (homogeneous enzymatic assay for the direct quantitative determination of HDL and LDL cholesterol). The intra- and inter-assay CV were <7% for all biochemical assays performed.

High-sensitivity CRP levels

After an overnight fast of at least 8 h, in the morning (8.00–10.00 a.m.), were collected the blood samples and subsequently stored at −80 °C until processed. In particular, through a method of particle-enhanced immunonephelometry using CardioPhase® (Siemens Healthcare Diagnostics, Marburg, Germany), were analysed serum hs-CRP levels. The interassay coefficient of variation was <10%. The lower detection limit of hs-CRP was 0.2 mg/L, the upper limit was 500 mg/L.

Cardio-Metabolic Indices

In according to Matthews et al. homeostatic model assessment-insulin resistance (HoMA-IR) was calculated and a value of >2.5 was used as cut-off of insulin resistance. Visceral adiposity index (VAI) score has been calculated using the following sex-specific formula: [WC/36.58 + (1.89 × BMI)] × (triglycerides/0.81) × (1.52/HDL), with triglycerides levels expressed in mmol/L and HDL levels expressed in mmol/L. Age-specific VAI cut-off values were used according to Amato et al. Fatty liver index (FLI) was calculated with the formula proposed by Bedogni G. et al [FLI = eL/(1 + eL) × 100, L = 0.953 × loge triglycerides + 0.139 × BMI + 0.718 × $\log_e \gamma$GT + 0.053 × WC−15.745]. A FLI value >60 indicated the presence of hepatic steatosis.

Body Composition and phase angle

The same device was used to avoid interdevice and interobserver variability. According to Kushner, before applying the electrodes (BIATRODES Akern Srl; Florence—Italy), PCOS patients were asked to remove their shoes and socks and the contact areas of electrodes on the hand and the ipsilateral foot, were scrubbed with alcohol immediately before their placement. The resistance (R) and reactance (Xc) data were obtained under strictly standardized conditions. In particular, all PCOS patients had refrained from exercising for six hours and eating and drinking within 24 h before testing, and were supine with limbs slightly spread apart from the body.
